# Accuracy of implant surgical guides fabricated using computer numerical control milling for edentulous jaws: a pilot clinical trial

**DOI:** 10.1186/s12903-020-01283-4

**Published:** 2020-10-21

**Authors:** Jinyou Chai, Xiaoqian Liu, Ramona Schweyen, Jürgen Setz, Shaoxia Pan, Jianzhang Liu, Yongsheng Zhou

**Affiliations:** 1 Department of Prosthodontics, Peking University School and Hospital of Stomatology, National Engineering Laboratory for Digital and Material Technology of Stomatology, Beijing Key Laboratory of Digital Stomatology, No. 22 Zhongguancun South Avenue, Haidian District, Beijing, 100081 China; 2 Department of Prosthodontics, Martin-Luther-University Halle-Wittenberg, Halle, Saale, Germany

**Keywords:** Accuracy, Guided implant surgery, Edentulous, Surgical template, CNC milling

## Abstract

**Background:**

To evaluate the accuracy of a computer numerical control (CNC) milled surgical guide for implant placement in edentulous jaws.

**Methods:**

Edentulous patients seeking implants treatment were recruited in this prospective cohort study. Radiographic guides with diagnostic templates were fabricated from wax-up dentures. Patients took cone-beam computed tomography (CBCT) wearing the radiopaque radiographic guides. Implant positions were virtually designed in the planning software based on the CBCT data, and the radiographic templates were converted into surgical guides using CNC milling technique. Forty-four implants were placed into 12 edentulous jaws following guided implant surgery protocol. Post-surgery CBCT scans were made for each jaw, and the deviations between the planned and actual implant positions were measured. Deviation of implant position was compared between maxilla and mandible, and between cases with and without anchor pins using independent *t*-test.

**Results:**

Nine patients (3 males and 6 females) with 12 edentulous jaws were recruited. The mean age of patients was 59.2 ± 13.9 years old. All 44 implants was placed without complication and survived, the mean three dimensional linear deviation of implant position between virtual planning and actual placement was 1.53 ± 0.48 mm at the implant neck and 1.58 ± 0.49 mm at the apex. The angular deviation was 3.96 ± 3.05 degrees. No significant difference was found in the deviation of implant position between maxilla and mandible (*P* = 0.28 at neck, 0.08 at apex), nor between cases with and without anchor pins (*P* = 0.87 at neck, 0.06 at apex).

**Conclusions:**

The guides fabricated using the CNC milling technique provided comparable accuracy as those fabricated by Stereolithography. The displacement of the guides on edentulous arch might be the main contributing factor of deviation.

*Trial registration*: Chinese Clinical Trial Registry, ChiCTR-ONC-17014159 (July 26, 2017).

## Background

Surgical templates for guided implant surgery have gained increasing importance in implant dentistry [1, 2]. The fabrication of implant surgical guides usually follows a digital workflow [3]: cone-beam computed tomography (CBCT)/multidetector computed tomography (MDCT) data are converted into a virtual, three-dimensional (3D) digital model using planning software, and this allows virtual implants to be placed in an ideal, prosthetically driven manner. The virtual implant position can then be recorded in a template for guided surgery.

Currently, there are two ways to fabricate surgical templates, namely, rapid prototyping (RP) and milling. Rapid prototyping or stereolithography (SLA) is the most widely used technique to fabricate surgical guides. Data from the Computed tomography (CT) scan, intraoral scan (or model scan) and digitized try-in prosthesis are fused. Double scan technique was commonly used. During the data fusion process, errors can be introduced into the system [4]. Linear RP processing error has been reported to be 0.22–0.24 mm [5]. For edentulous jaws, data fusion to fabricate a surgical guide is even more challenging due to the lack of rigid support. The accuracy of the SLA surgical guide is influenced by parameter settings and calibration algorithm used in the planning software. Stumpel reported that the mean difference between the SLA duplicate denture printed from Digital Imaging and Communications in Medicine (DICOM) data and the original diagnostic prosthesis ranges from 0.56 to 2.17 mm [6].

Milled implant surgical templates are made based on coordinate alignment [7]. A radiopaque radiographic template was fabricated from wax-up denture. Patients took CBCT wearing the radiographic template, and the radiopaque dentition and the bone structure could be viewed in one CT scan. No data fusion was needed. The radiographic guide was then converted into a surgical guide by milling slots for he guide sleeves [8]. Errors caused by the fusion of multisource data can be avoided.

For laboratory milled surgical guide, the most critical step is to transfer the virtual implant position into the coordinates of the milling machine. Previous studies have reported several transfer methods, such as the “X-cube”, orthogonally designed acrylic rods or a standard template [9, 10]. In 2018, a kind of implant surgical guide fabricated using 5-axis Computer Numerical Control (CNC) milling machine for guided implant placement in the edentulous jaw was introduced [11]. The laboratory-based computer aided design and computer aided manufacturing (CAD–CAM) system provides a digital workflow in which only two steps involve manual interventions. The first step is the fabrication of the acrylic base for the radiographic template. The second step is the fixation of metal sleeves in the sleeve slots. Coordinates synchronization can be realized using a specially designed diagnostic template.

Many studies have investigated the accuracy of stereolithography surgical guides for edentulous jaws (Table 1). However, there were only a few studies addressing the accuracy of the laoratory based CNC milled surgical guide. Chai et al. reported the preclinical fabrication accuracy of the CNC milled surgical guide as 1.06 mm at the neck and 1.12 mm at the apex [11]. Park reported the technical deviation of the milled template was 0.68 mm horizontally, and 0.41 mm vertically [7]. There was no study reporting the clinical accuracy of the CNC milled surgical guide for edentulous jaws.

**Table 1:** Accuracy of implant placement using mucosa-supported surgical guide for edentulous jaws reported in previous studies

References	Linear deviation (mm)		Angular deviation (degree)
	Implant neck	Implant apex	
Albiero et al. [13]	1.28 ± 0.6	1.65 ± 0.71	3.42 ± 1.52
Vercruyssen et al. [14]	0.9	1.2	2.7
Geng et al. [25]	0.69 ± 0.66	0.94 ± 0.75	2.71 ± 2.58
Sun et al. [26]	1.48 ± 0.96	NA	4.05 ± 3.07
Vercruyssen et al. [27]	1.38 ± 0.64	1.6 ± 0.7	2.71 ± 1.36
Vercruyssen et al. [27]	1.23 ± 0.6	1.57 ± 0.71	2.86 ± 1.6
Cassetta et al. [28]	1.68 ± 0.6	2.19 ± 0.83	4.67 ± 2.68
Arisan et al. [23]	0.81 ± 0.32	0.87 ± 0.32	3.47 ± 1.14
Cassetta et al. [29]	1.49 ± 0.63	1.9 ± 0.83	3.93 ± 2.34
Cassetta et al. [29]	1.55 ± 0.59	2.05 ± 0.89	5.46 ± 3.38
Di Giacomo et al. [30]	1.35 ± 0.65	1.79 ± 1.01	6.53 ± 4.31
D'Haese et al. [21]	0.91 ± 0.44	1.13 ± 0.52	2.6 ± 1.61
Pettersson et al. [31]	0.95 ± 0.55	1.22 ± 0.63	2.76 ± 1.76
Arisan et al. [32]	1.24 ± 0.51	1.4 ± 0.47	4.23 ± 0.72
Ozan et al. [33]	1.06 ± 0.6	1.6 ± 1.0	4.51 ± 2.1
Ersoy et al. [34]	1.28 ± 0.92	1.6 ± 1.08	5.1 ± 2.59

Therefore, the aim of this study was to evaluate the accuracy of implants placed using a CAD–CAM fabricated CNC-milled implant surgical guide in edentulous jaws. The null hypothesis was that by using the CAD–CAM fabricated CNC-milled implant surgical guides, the position differences between the virtually planned and actually placed implants would be comparable to those of SLA surgical guides reported in the literature.

## Methods

### Participants

The patients who were edentulous in one or in both jaws and seeking implant-supported prosthesis treatment in the Department of Prosthodontics at Peking University School and Hospital of Stomatology from December 2017 to June 2018 were included in this study. The inclusion and exclusion criteria are shown in Table 2. The study was reviewed and approved by the Institutional Review Board of Peking University School and Hospital of Stomatology. The study was registered in the Chinese Clinical Trial Registry (ChiCTR-ONC-17014159). This study was undertaken with the understanding and written informed consent of each individual participant and was conducted in accordance with the World Medical Association’s Declaration of Helsinki (Version, 2013).

**Table 2:** Inclusion and exclusion criteria

Inclusion criteria	Exclusion criteria
Age > 20 years old	In need of complicated bone augmentation procedure
Being edentulous for more than 6 months	Local or systemic contraindication for implant therapy (i.e. uncontrolled diabetes, hemophilia, metabolic bone disorder, history of renal failure, radiation treatment to the head or neck region, current chemotherapy, and pregnancy etc.)
Willing to receive implant treatment	Smoking more than 10 cigarettes per day
	Buccal-lingual width of keratinized tissue less than 6 mm

### Design and fabrication of the radiographic template

**Fig. 1:**
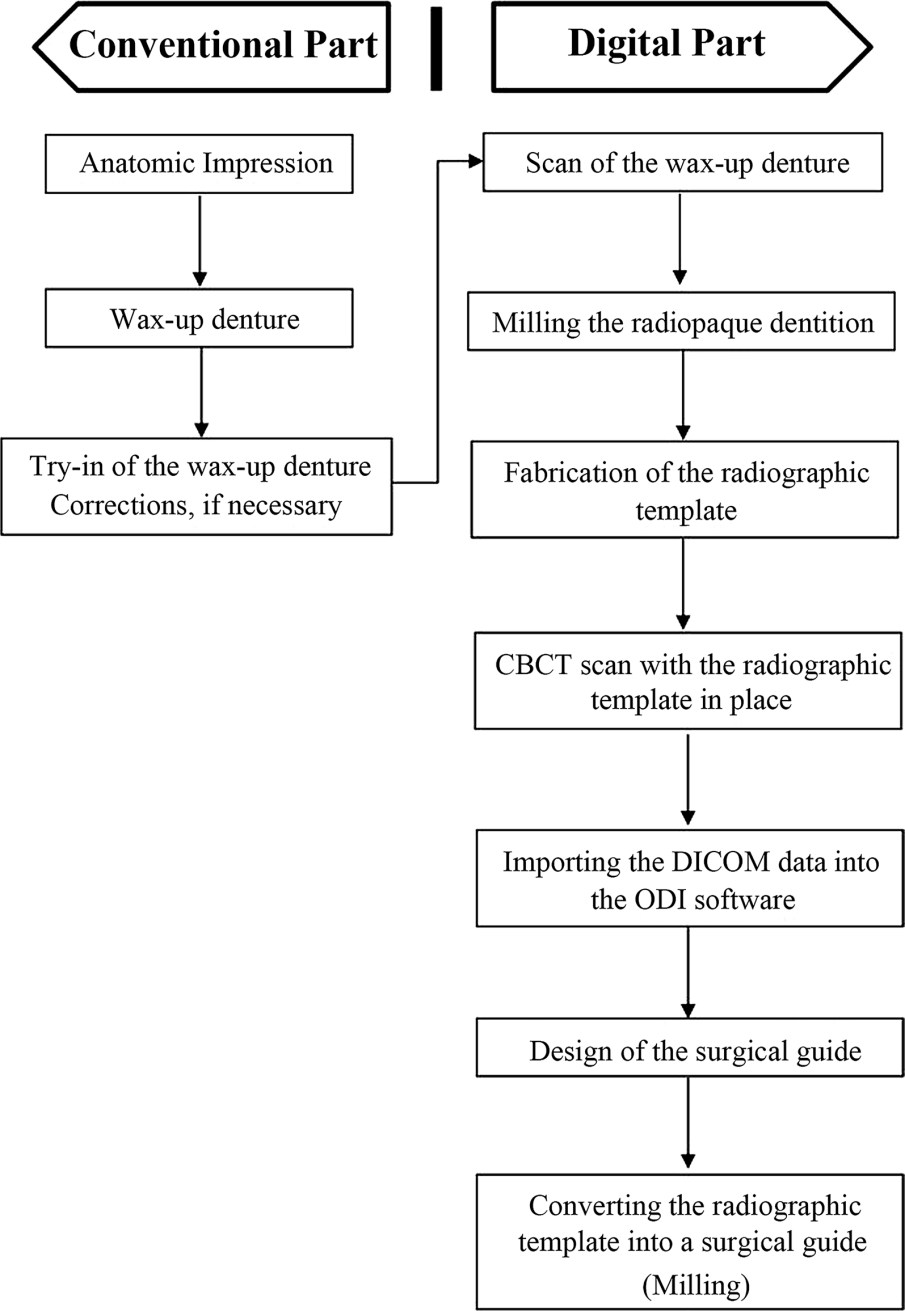
Workflow of the conventional and digital parts of the guide fabrication procedure

**Fig. 2:**
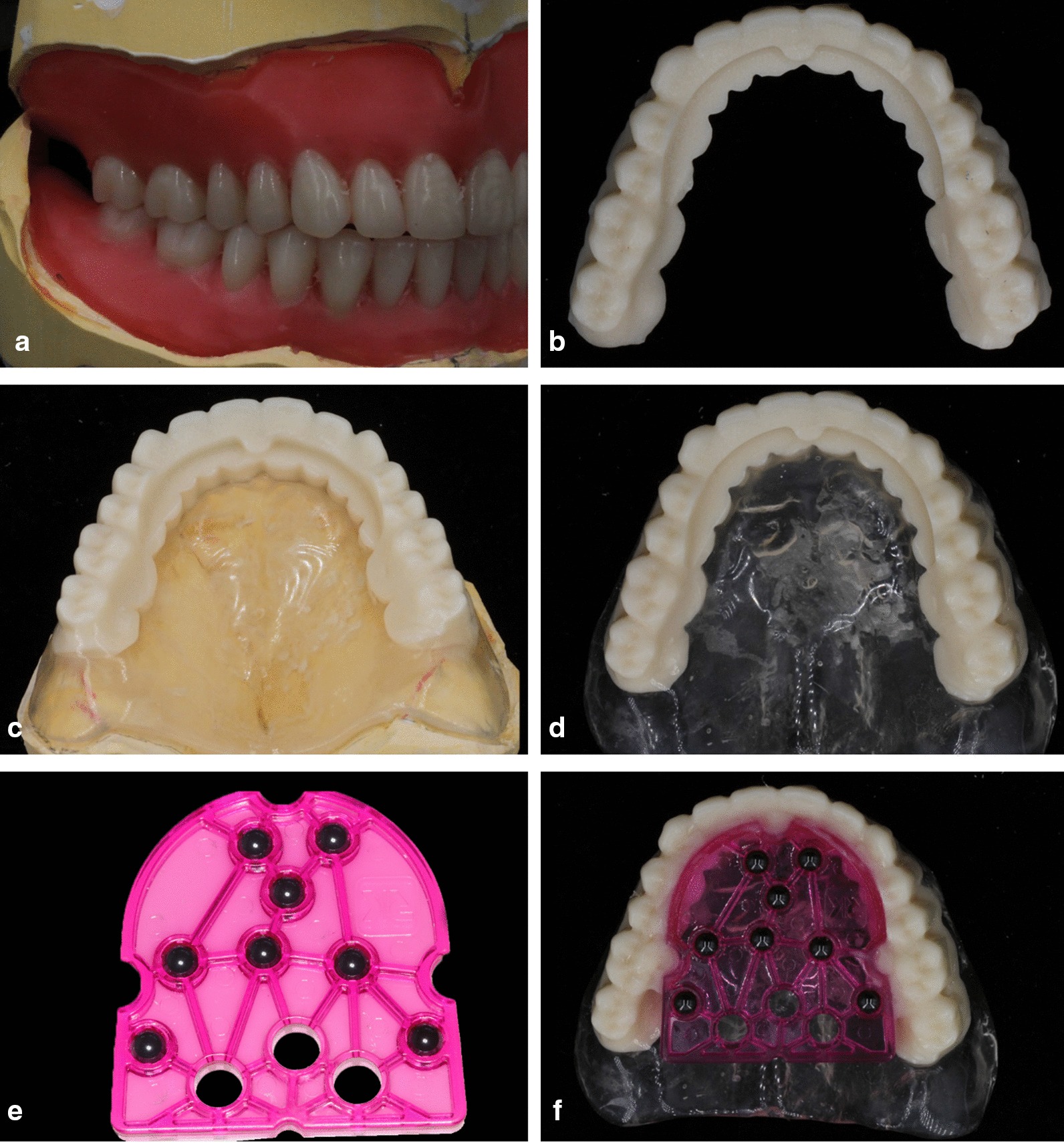
The radiographic template. **a** The wax-up on the maxillary and mandibular edentulous models. **b** Radiopaque PMMA dentition duplicated from the digitized wax-up. **c** Translucent resin base for radiopaque dentition built on the stone model. **d** Radiopaque dentition with the transparent resin base removed from the stone model. **e** Diagnostic template with eight zirconia beads. **f** The finished radiographic template

The study protocol is summarized in Fig. 1. For each patient, after conventional impressions were taken for both the maxilla and mandible, stone models were fabricated. Maxillomandibular relationship registration was performed and verified, and the models were mounted in an articulator using facebow transfer technique. The wax-up dentures (Fig. 2a) were tried in the patient’s mouth, and after clinically necessary corrections, the wax-up dentures were sent back to the laboratory for the production of radiographic templates.

The wax-up was digitized using a table-top scanner (D2000, 3shape, Copenhagen K, Denmark). A radiopaque polymethyl methacrylate (PMMA) blank (Organical^®^ PMMA, Organical, Berlin, Germany) was used to mill the dentitions on a 5-axis CNC milling machine (Fig. 2b, Organical^®^ Multi S, Organical, Berlin, Germany). A transparent self-cure resin base was built on the model (Fig. 2c, d). Finally, a registration template (Diagnostic Template, Organical^®^, Berlin, Germany) was bonded to the lingual side of the radiopaque dentition to complete the radiographic template (Fig. 2e, f).

### CBCT scan and virtual implant planning

**Fig. 3:**
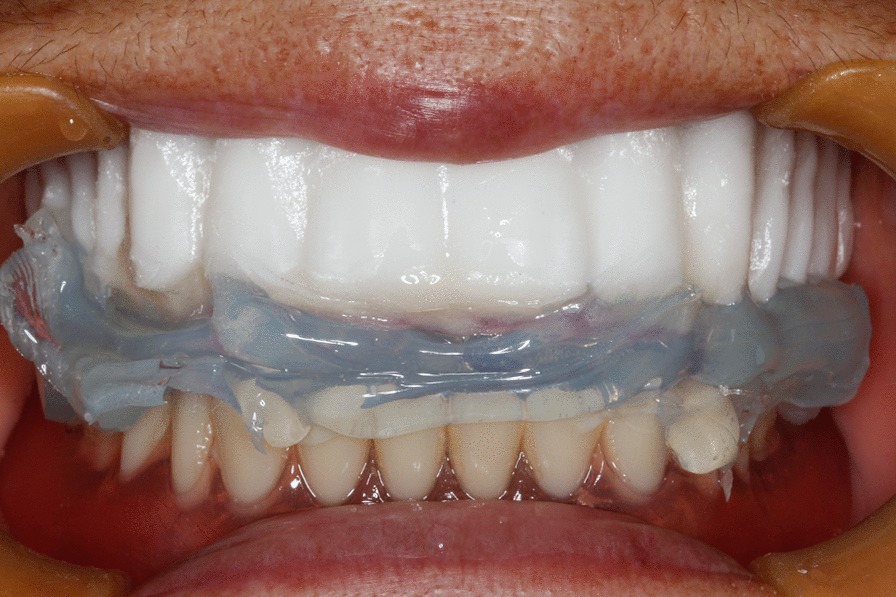
Radiographic template was tried in the patient’s mouth with the silicon index between the upper and lower arches. The patient underwent a CBCT scan while wearing the radiographic template

**Fig. 4:**
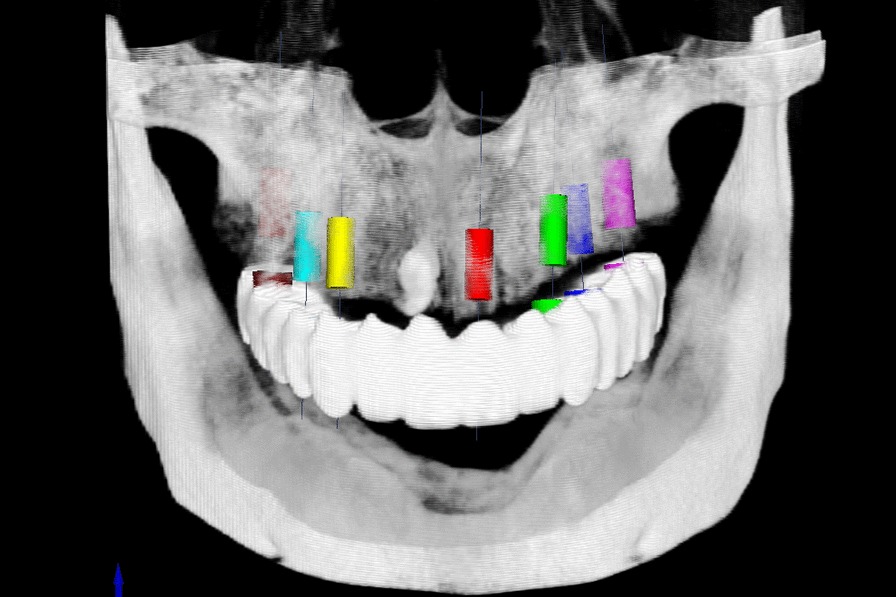
Virtual design of the implant positions in the virtual planning software (Organical^®^ Dental Implant, ODI 1.1.0.5, Organical, Berlin, Germany)

The radiographic template was placed in patient’s mouth to confirm its fit. A silicone index was made to further stabilize the template,. With the templates in the patient’s mouth, a CBCT scan (VGi, New Tom, Verona, Italy, voxel size 0.25 mm^3^, field of view 12 cm × 8 cm, voltage 110 kV, tube current 3.5 mA) was made (Fig. 3). The image data were exported in the DICOM format and exported to virtual planning software (Organical^®^ Dental Implant, ODI 1.1.0.5, Organical, Berlin, Germany). The radiopaque dentitions and the alveolar bone could be viewed in the software (Fig. 4).

**Fig. 5:**
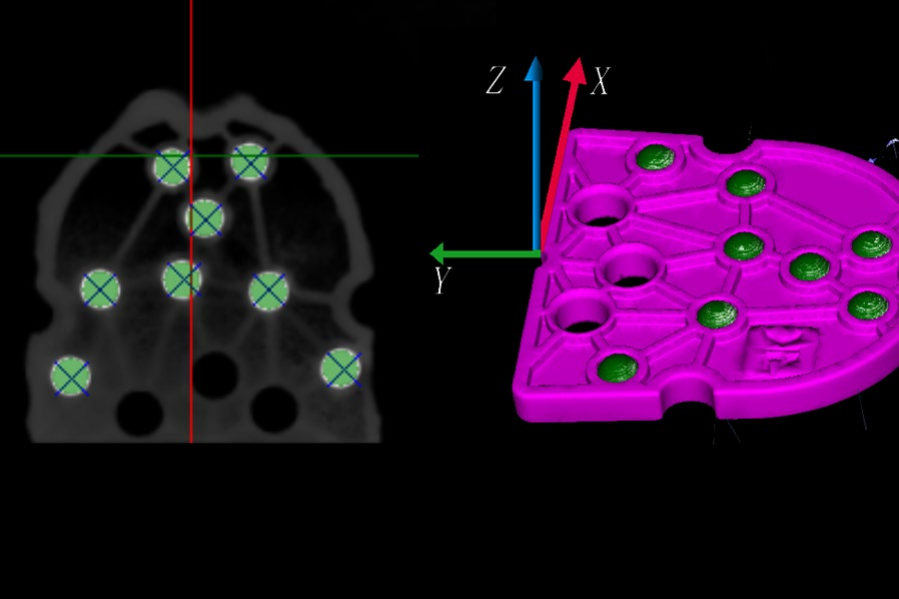
Coordinate alignment in the implant planning software. Left: The software identified the zirconia beads in the diagnostic plate in the image. Right: The positions of the diagnostic plate and the radiographic template were aligned with the coordinates of the software

Virtual implant planning was conducted in the software in accordance with the prosthetic treatment protocol (Fig. 4). The software aligned the spatial coordinates of the radiographic templates with its system coordinates by identifying the zirconia beads in the diagnostic plate in the DICOM data (Fig. 5). The position information of the virtually designed implants was then transferred into coordinate values that could be identified by the milling software. The planning data were then exported in the initial graphics exchange specification (IGES) format.

### Milling of surgical guides

**Fig. 6:**
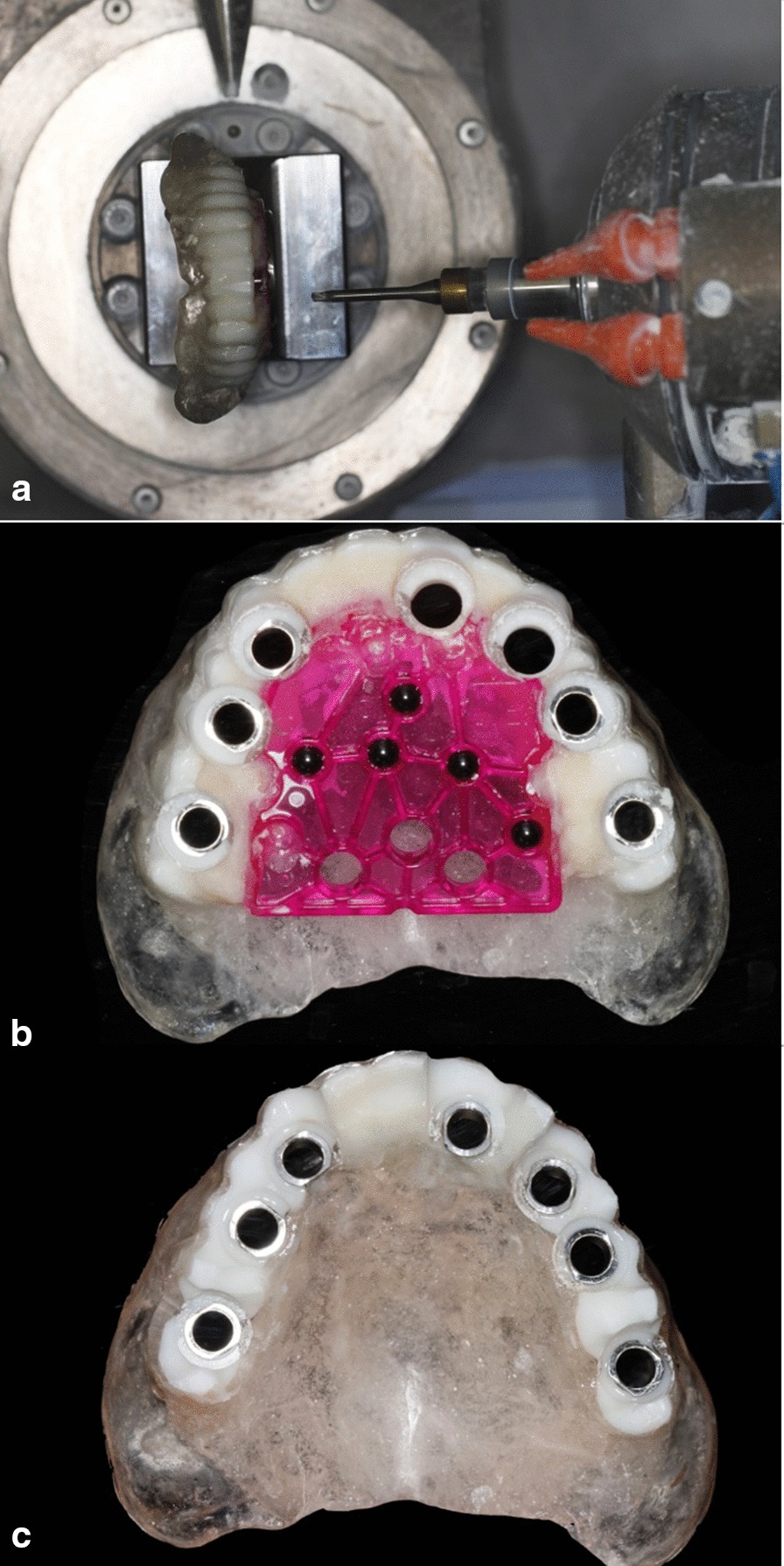
The radiographic template was transferred into the surgical guide by the CNC milling process. **a** The radiographic template was fixed on the holder of the CNC milling machine, and slots of the sleeves were milled on the radiographic template. **b** Steel guide sleeves were installed into the slots. **c** The registration template was removed, and the radiographic template was transferred into a surgical guide

The implant planning data were transferred into the milling software (Organical^®^ Mill2, Organical, Berlin, Germany), and the radiographic guide with the diagnostic template was fixed on the 5-axis CNC machine (Organical^®^ Multi S, Organical, Berlin, Germany). Slots for the guide sleeve of each implant were milled on the radiographic templates. The guide sleeves were precisely installed into the slots (Fig. 6). Thus, the radiographic template was transferred into an implant surgical guide.

### Guided surgery

All the surgeries were performed by two experienced dentists (S.P and J.L) at the Department of prosthodontics, Peking University School and Hospital of Stomatology. Before surgery, the surgical guide and the silicon index were disinfected in 0.12% chlorhexidine for 30 min. The surgical guide was positioned on the edentulous jaw with the interocclusal silicon index to confirm proper seating.

After local anaesthesia, the surgical guide was either fixed on patient’s alveolar ridge by three lateral fixation pins or retained using fixation anchors through guide sleeves after the first twist drill. A punch drill was used to remove the mucosa on top of the alveolar ridge, and a flapless guided implant placement protocol was followed.

**Fig. 7:**
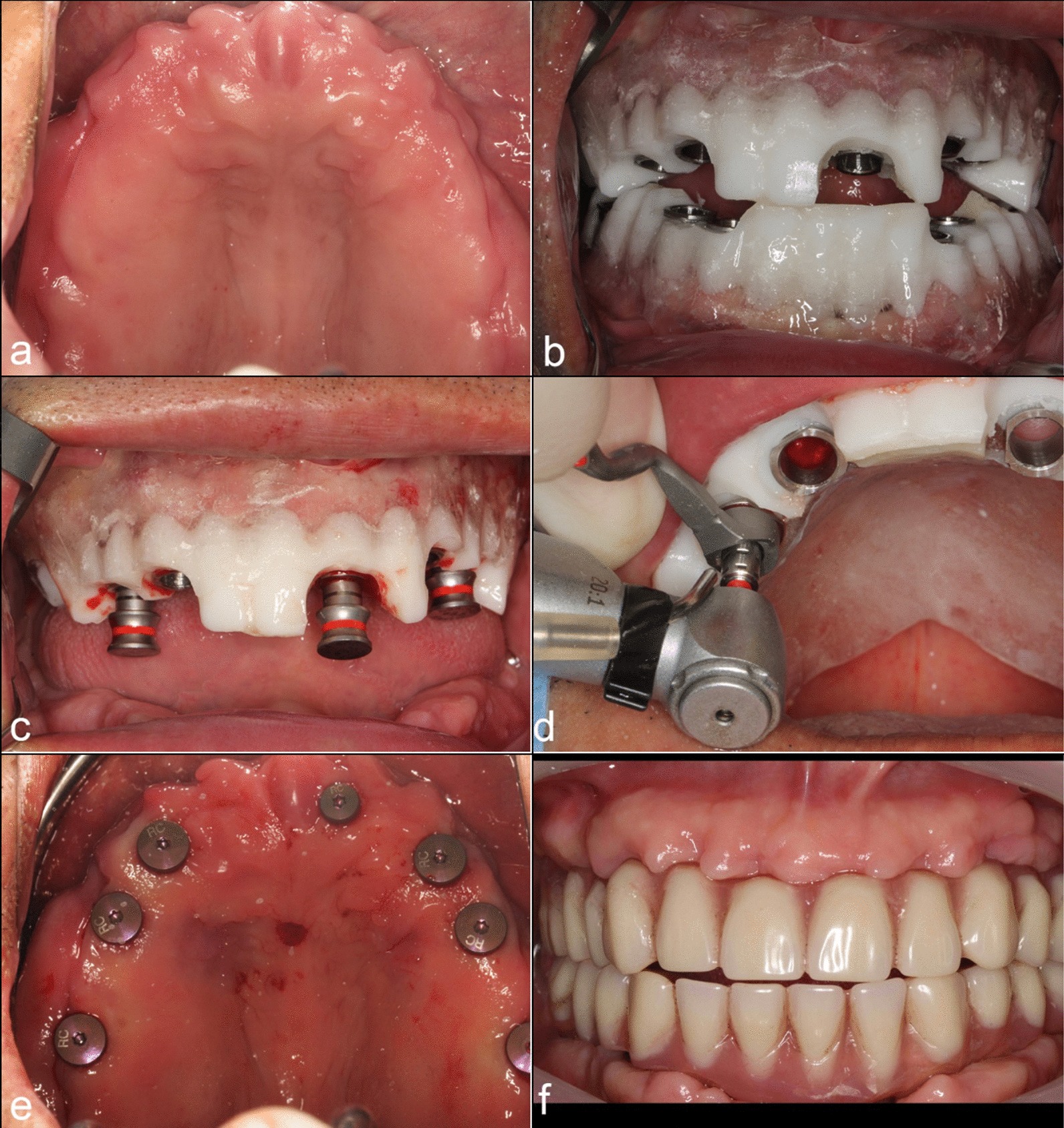
Guided surgery using CNC-milled mucosa-supported guide. **a** Edentulous maxilla with sufficient keratinized gingiva. **b** The upper and lower surgical guide. **c** The guide was fixed on the edentulous arch by fixation anchors through the guide sleeves. **d** Osteotomy was performed using drill handles and guided instruments. **e** Seven implants were placed following a flapless protocol. **f** Screw-retained immediate fixed prosthesis modified from a previous complete denture

Based on the virtual planning, the correct combination of drill handles and guided instruments was used for osteotomy site preparation, and the implants were installed (Bone level implant or Tissue level implant, Institut Straumann AG, Switzerland, Fig. 7). Guided bone regeneration (GBR) procedure was performed for one implant with bone graft material (0.25 g, Bio-Oss^®^, Geistlich, Switzerland) and collagen membrane (Bio-Guide^®^, Geistlich, Switzerland).

### Deviation measurement

**Fig. 8:**
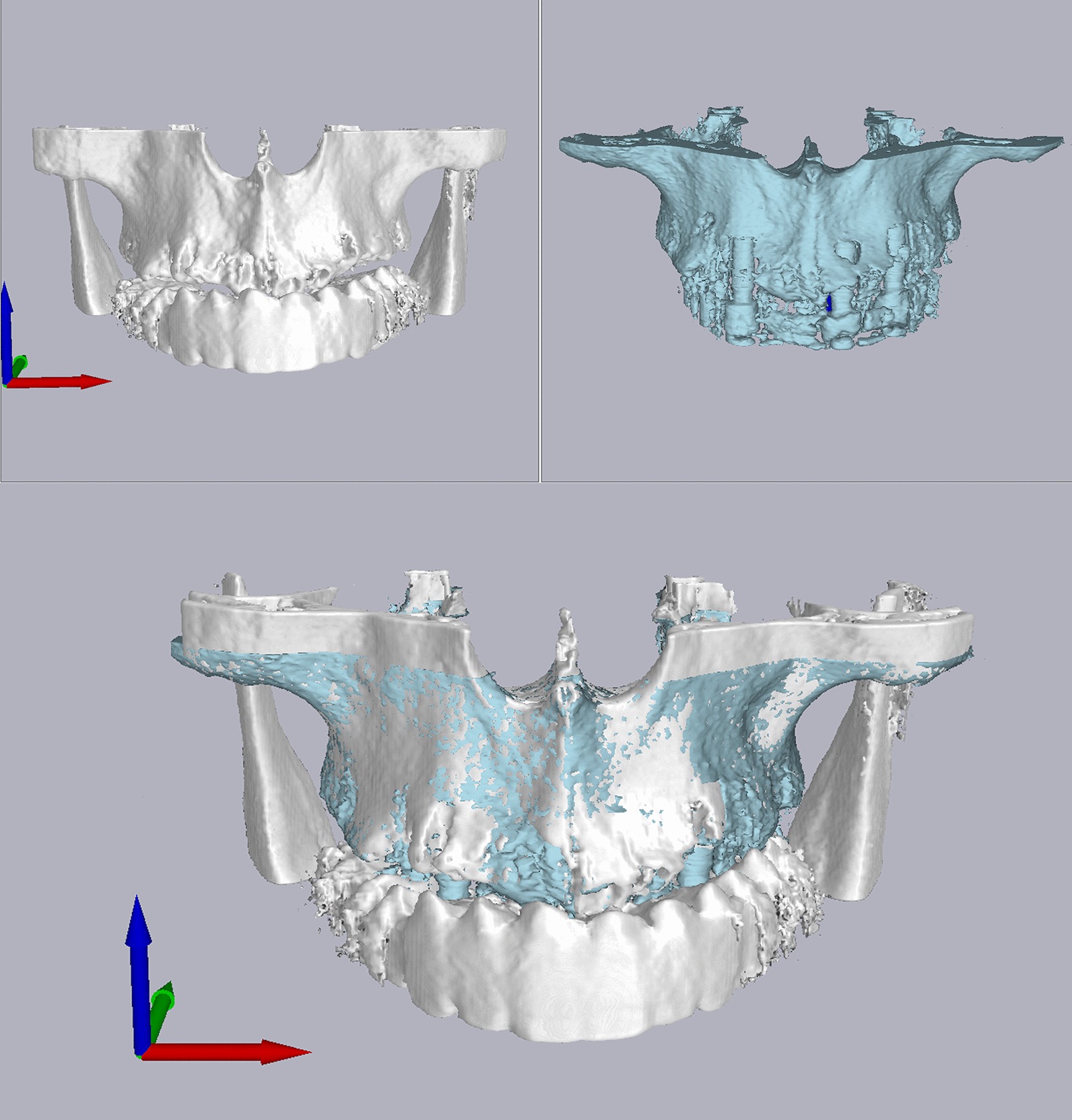
The superimposition of the pre- and post-operative data reconstructed from the CBCT scans

After implant placement, a second CBCT scan was made for each patient. The post-surgical CBCT data were imported into Mimics (Mimics 19.0, Materialise, Leuven, Belgium), and post-operative digital models in the Standard Tessellation Language (STL) format were generated from the DICOM data. A 1.25 mm layer of bone around the implant was removed using the Masks, Morphology, and Boolean function in the software. Finally, the data of the bone structure of the edentulous maxilla and mandible together with the isolated implants in the STL format were exported from Mimics software and imported into the virtual planning software, where the data were superimposed with the pre-surgical CBCT image that contained the virtually planned implants (Fig. 8).

The deviation between the virtually planned and actually placed implant positions was measured at the neck and apex of each implant.

Four parameters were defined, namely, the global deviation, horizontal deviation, depth deviation, and angular deviation. All parameters, except for the angular deviation, were measured both at implant neck and apex.

**Fig. 9:**
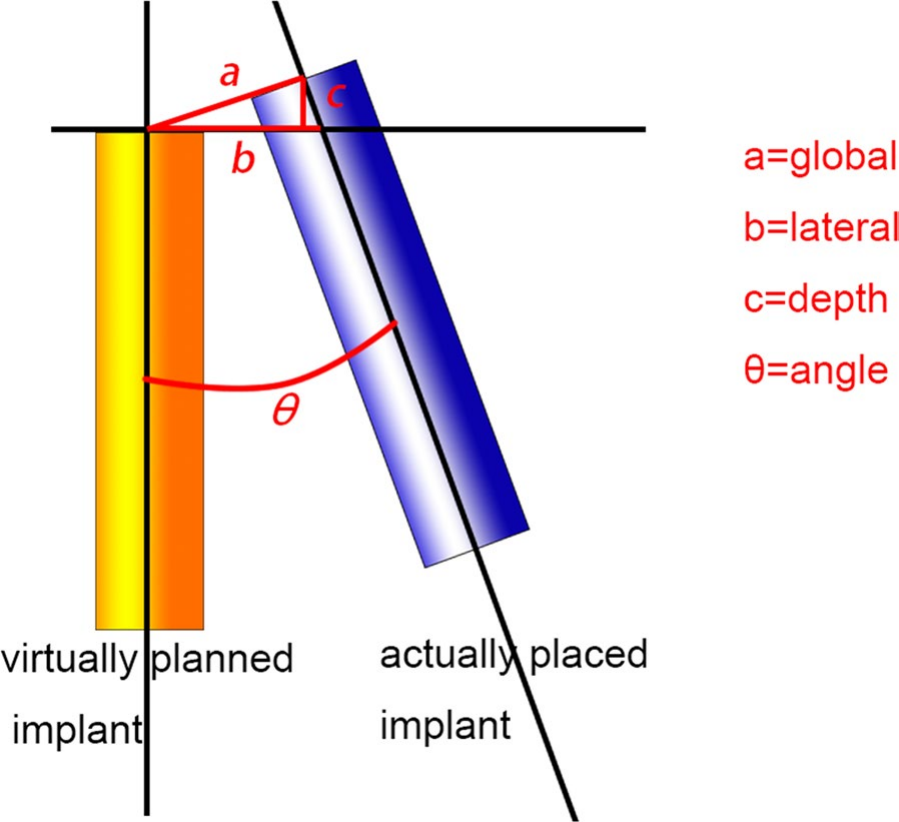
Evaluation of the three-dimensional deviation between the planned and actual implant position

The global deviation was defined as the 3D distance between the centres of the neck (or apex) of the corresponding virtually planned and actually placed implants. To calculate the lateral deviation, a plane perpendicular to the longitudinal axis of the planned implant and through its coronal or apical centre was defined and set as the reference plane. The horizontal deviation was defined as the distance between the coronal (or apical) centre of the planned implant and the point of intersection of the longitudinal axis of the placed implant with the reference plane. The depth deviation was defined as the distance between the coronal (or apical) centre of the placed implant and the reference plane. The angular deviation was defined as the three-dimensional angle between the longitudinal axis of the planned and placed implants (Fig. 9).

To analyse the factors contributing to implant deviation, the mean global deviation in the maxillary cases and that in the mandibular cases were compared. Mean global deviation in cases with and without lateral fixation pins were also compared.

#### Mean difference of inter-implant distance

**Fig. 10:**
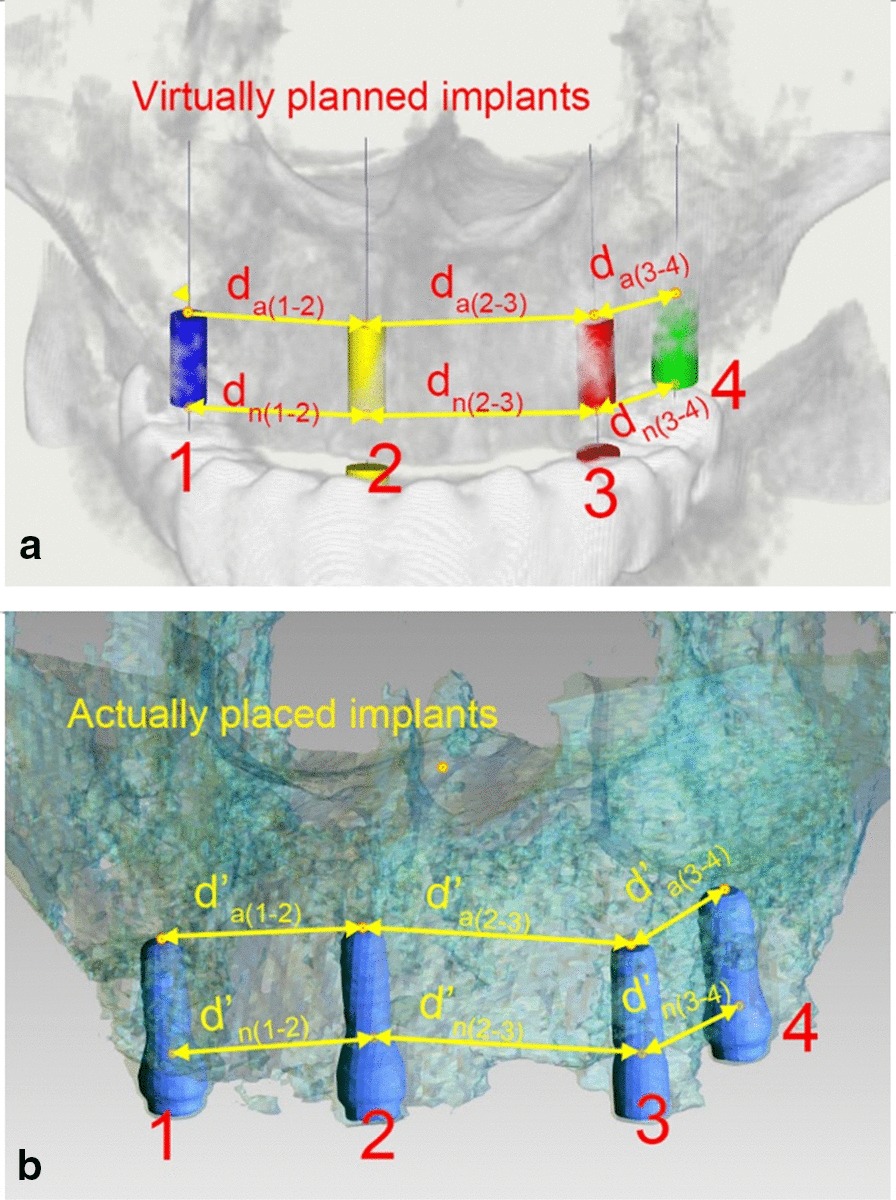
a, The inter-implant distance at the neck level of two neighbouring implants in the virtual planning model was named d_n_, and that at the apex level was named d_a_; b, The inter-implant distance at the neck level of two neighbouring implants in the post-operative CBCT scan was named d_n_′, and that at the apex level was named d_a_′; The mean difference between the virtual and actual inter-implant distances of two adjacent implants was calculated

The distances between each pair of neighboring implants were measured in the virtual planning and the actual post-surgery CBCT scans. The mean differences between the virtual and actual inter-implant distance at implant neck and apex area were calculated, this will represent the random errors in the surgical template (Fig. 10).

### Data analysis

The data were analysed descriptively using statistical software (IBM SPSS Statistics, v20.0; IBM Corp, Chicago, IL, USA). To determine the contributing factors for the deviation in implant position, the deviation values between the upper and lower jaws as well as between cases using fixation pins for the surgical guide and cases not using fixation pins were compared using independent *t*-tests.

## Results

Nine patients with 12 edentulous jaws were recruited in this prospective cohort study. The mean age of the patients was 59.2 ± 13.9 years old. The patients’ demographic data and distribution of the implants are summarized in Table 3. A total of 44 implants were placed using CAD–CAM CNC-milled guides. During the surgery and with an average post-surgery follow-up time of 6 months, no post-operative complications, such as haemorrhages, sinus pathologies, severe pain, or inflammation, were recorded. The 6 months post-operative survival rate of the implants were 100%.

**Table 3:** Patients’ demographic information and implant distribution

	Number (percentage)
Patients	
Male	3 (33.3%)
Female	6 (66.7%)
Edentulous arches	
Upper	7 (58.3%)
Lower	5 (41.7%)
Implants	
Upper	21 (47.7%)
Lower	23 (52.3%)

Lateral fixation pins were used in three surgical templates with 14 implants placed, and the other nine surgical guides were retained with fixation anchors through the guide sleeves after the first twist drill was used for the osteotomy. The average global deviation at the implant shoulder was 1.53 ± 0.48 mm, and that at the apex was 1.58 ± 0.49 mm. The mean angular deviation was 3.96 ± 3.05 degrees. The global, horizontal and vertical deviations are shown in Table 4. No significant differences were found between the global coronal deviation and the global apical deviation. The horizontal deviation was significantly larger than the depth deviation at both the implant neck and apex (*p* < 0.05).

**Table 4:** Deviation between the virtually planned and actually placed implant positions

	Mean	SD	Min	Max
Linear deviation at the implant neck (mm)				
Horizontal	1.28	0.40	0.42	2.36
Depth	0.67	0.58	0.01	2.20
Global	1.53	0.48	0.61	2.58
Linear deviation at the implant apex (mm)				
Horizontal	1.28	0.53	0.13	2.19
Depth	0.70	0.58	0.01	2.21
Global	1.58	0.49	0.47	2.74
Angular deviation (degrees)	3.96	3.05	0.60	16.72

The effects of jaw position and lateral fixation pin on the deviation between the planned and actual implant positions were evaluated (Table 5). There was a trend showing that the mean global deviation in implant position in the maxilla was lower than that in the mandible at both implant neck and apex. However, the difference was not statistically significant (*p* = 0.280 for the value at the implant neck, *p* = 0.084 for the value at the implant apex). No significant difference was found in the implant deviation between the surgical guides with and without lateral fixation pins.

**Table 5:** Global deviation (mm) between the virtually planned and actually placed implant positions in the different subgroups

Global deviation	Edentulous arch			Lateral fixation pins		
	Upper (n = 7)	Lower (n = 5)	*p* value	With	Without	*p* value
Neck	1.45	1.61	0.28	1.51	1.54	0.87
Apex	1.44	1.70	0.08	1.81	1.47	0.06

Inter-implant distance for every two neighboring implants were measured in both the virtual planning CBCT and post-operative CBCT, and the difference of inter-implant distance between pre- and post-surgical CBCT data were calculated. The mean difference of the inter-implant distance was 0.48 ± 0.51 mm at the implant neck and 0.50 ± 0.43 mm at the implant apex. The minimum value was 0.04 mm coronally and 0.02 mm apically. The maximum value was 2.71 mm coronally and 1.81 mm apically.

## Discussion

In this study, the accuracy of a CAD–CAM CNC-milled implant surgical template for edentulous jaws was investigated. The mean deviation between virtually planned and actual placed implant positions was 1.53 ± 0.48 mm in the coronal plane and 1.58 ± 0.49 mm in the apex area. These results are comparable to those of previous studies that reported the accuracy of SLA surgical guides for implant placement in edentulous jaws. The null hypothesis was not rejected.

In some previous studies [12–14], stereolithography (SLA) surgical guides were evaluated. Seo et al. [15] reported the accuracy of SLA mucosa-supported surgical guides for edentulous jaws. The mean coronal deviation was less than 1.68 mm, and the mean apical deviation was less than 2.19 mm. Few studies have investigated the accuracy of CNC-milled surgical guides for edentulous patients. The accuracy of the milled surgical guide from the present study is acceptable for clinical use. However, the factors that contribute to the deviation in implant position should be identified.

For the RP solution, the guide was designed with the digital model on the computer and fabricated by 3D printing techniques [16]. Data fusion was performed to provide clinicians with necessary information in digital form. Errors might be introduced into the final template during the registration of multiple data [4, 17]. For example, the fusion of DICOM data extracted from the CBCT of edentulous patients wearing diagnostic prostheses and the data from the low-dose CBCT of the prosthesis is based on marker registration. Its accuracy is influenced by the number of markers used and their locations. The accuracy of the RP surgical template also depends on the 3D reconstruction of the marked denture. An excessively high or low grey value threshold will result in a surgical template that is too thin or too thick [17].

For the workflow in our study, all necessary data were acquired by one CBCT scan, and no intraoral or model scans were needed. The radiographic template was made in the dental laboratory with radiopaque material, and its base was constructed directly on the stone model to guarantee a precise fit. The CBCT scan was taken with the radiographic template in place, thereby providing data of the future dentition as well as the alveolar bone. Dual CBCT image fusion [18] was not needed, and errors generated in this procedure could be avoided. Another innovative design of this system is the diagnostic template. It provides both the spatial registration of the CBCT data for the design software and the positioning holes for the alignment of the coordinate systems between the radiographic template and the CNC milling machine. By mean of the diagnositc template, the planning information was transferred to the final surgical guides. Compared with previously reported milling technique [9, 19, 20], the fabrication process of the surgical template comprised mostly of the digital workflow and reduced manual work to a current minimum. The technique introduced in this study uses CAD–CAM in most of the workflow, thereby simplify the process and produce surgical templates with an accuracy comparable to that of templates made with the RP technique. However, it should be noted that the radiographic template was fabricated on the stone model, and the expansion of the stone may result in difference between the model and the real oral cavity.

The mean difference between the virtual and actual inter-implant distance was significantly smaller than the mean global deviation for a single implant in both the coronal and apical areas. When the inter-implant distances were calculated, inherent systematic error was eliminated, while for a single implant, the deviation included systematic error. Only production errors and errors occurring during the surgical procedure were related to the inter-implant distance deviations [21]. The positioning error of the surgical template may be one of the principal contributing factors to the systematic error [21].

**Fig. 11:**
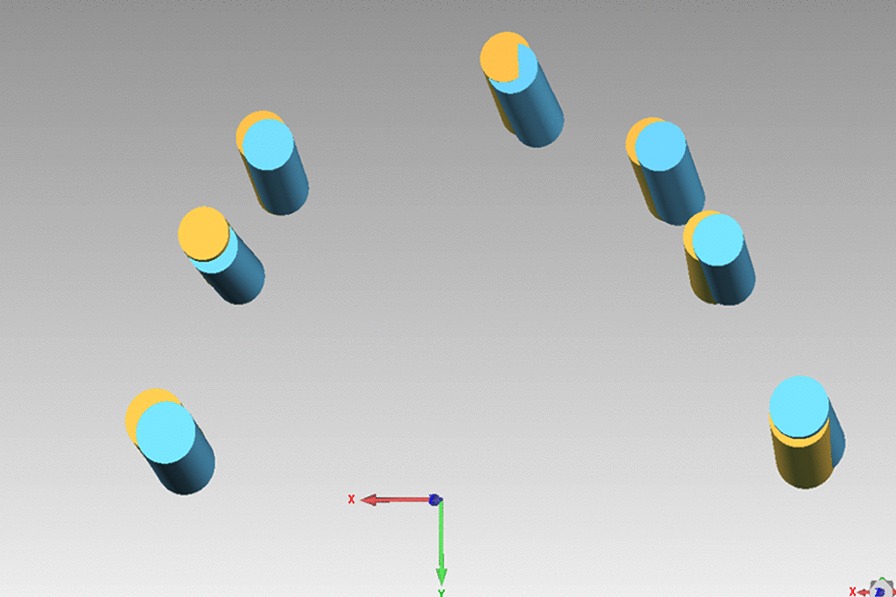
The virtually planned (yellow cylinder) and actually placed implants (blue cylinder) were superimposed, and it can be observed that the actual implants were shifted towards the same direction, indicating a shift of the surgical guide during surgery (systemic error)

Surgical template repositioning on the edentulous arch can be challenging due to the resilient nature of the mucosa covering edentulous jaws. There is no rigid support for the template, and anaesthesia during surgery can also lead to changes in the position of the surgical guide. This can also be observed in the accuracy evaluation. The planned and actual implants were superimposed, and the actual implants were shifted towards the same direction, indicating a shift in the surgical guide during surgery (Fig. 11).

To reduce the effect of edentulous mucosa, some authors [21] have suggested enlarging the base of the guides. Some studies have shown that the deviation of the implants in the maxilla is smaller than that in the mandible. In the present study, the absolute value of mean deviation in the implant position was smaller in the maxilla than in the mandible. However, no significant difference was found.

Some researchers have recommended anchor pins to fix the guides [22, 23]. However, no difference of deviation was found in this study between cases with anchor pins and those without. This finding was similar to a prior study [24] in which Verhamme et al. found no significant difference in guide accuracy between cases using anchor pins and cases without anchor pins.

There are several limitations in the present study. First, this study investigated only the accuracy of CNC-milled implant surgical templates for edentulous jaws. Second, the limited sample size of the implants in this study may not be sufficient to detect differences between subgroups of patients, and the findings should be interpreted carefully. Third, the cost of the milled surgical guide especially manpower invested in the guide fabrication procedure was not reported, and not compared with that of the SLA technique.

## Conclusions

With the limitation of the present study, one can concluded that the guides fabricated using the CAD–CAM CNC milling technique provided comparable accuracy as those fabricated by Stereolithography. The displacement of the guides on edentulous arch might be the main contributing factor of deviation. Clinical trials with larger sample size are needed.

## Data Availability

The datasets used and/or analysed during the current study are available from the corresponding author on reasonable request.

## Abbreviations

CNC: Computer numerical control
CBCT: Cone-beam computed tomography
MDCT: Multidetector computed tomography
3D: Three-dimensional
RP: Rapid prototyping
SLA: Stereolithography
CT: Computed tomography
CAD–CAM: Computer aided design and computer aided manufacturing
PMMA: Polymethyl methacrylate
IGES: Initial graphics exchange specification
GBR: Guided bone regeneration
STL: Standard tessellation language
